# Neurotransmitter Metabolic Disturbance in Methamphetamine Abusers: Focus on Tryptophan and Tyrosine Metabolic Pathways

**DOI:** 10.3390/toxics12120912

**Published:** 2024-12-16

**Authors:** Xi Wang, Weilan Wu, Jing Liu, Miaoyang Hu, Jie Cheng, Jianping Xiong, Xufeng Chen, Rong Gao, Jun Wang

**Affiliations:** 1 Center for Global Health, School of Public Health, Nanjing Medical University, Nanjing 211166, China; 2 Key Laboratory of Modern Toxicology (NMU), Ministry of Education, School of Public Health, Nanjing Medical University, 101 Longmian Avenue, Nanjing 211166, China; 3 Department of Hygienic Analysis and Detection, Key Laboratory of Modern Toxicology (NMU), Ministry of Education, School of Public Health, Nanjing Medical University, 101 Longmian Avenue, Nanjing 211166, China; 4 Department of Emergency Medicine, the First Affiliated Hospital of Nanjing Medical University, 300 Guangzhou Road, Nanjing 210029, China; cxfyx@njmu.edu.cn

**Keywords:** methamphetamine, neurotransmitters, tryptophan metabolism, tyrosine metabolism

## Abstract

Methamphetamine (METH) abuse disrupts the homeostasis of neurotransmitter (NT) metabolism, contributing to a wide range of neurological and psychological disorders. However, the specific effects of METH on NT metabolism, particularly for the tryptophan (TRP) and tyrosine (TYR) metabolic pathways, remain poorly understood. In this study, serum samples from 78 METH abusers and 79 healthy controls were analyzed using Ultra-High-Performance Liquid Chromatography with Tandem Mass Spectrometry (UHPLC-MS/MS). A total of 41 substances, primarily from the TRP and TYR metabolic pathways, were detected and subjected to multivariate analysis. Principal Component Analysis (PCA) and Partial Least Squares Discriminant Analysis (PLS-DA) revealed a significant separation of serum metabolites between METH abusers and controls, encompassing the disturbance of serotonergic, kynurenic, and microbial metabolism. In the serotonergic pathway, METH significantly reduced melatonin (MLT) levels and impaired the conversion of serotonin (5-HT) to N-acetylserotonin (NAS), a key precursor of MLT. In the kynurenic pathway, METH promoted a shift to the toxic metabolic pathway, evidenced by elevated levels of 3-hydroxykynurenine (3-HK) and quinolinic acid (QA). Furthermore, microbial metabolic pathway-related indole and its derivatives were markedly suppressed in METH abusers. Gender-specific differences were also observed, with NT metabolism in TRP and TYR pathways showing more pronounced alterations in male or female subgroups. Therefore, the current study provides a comprehensive overview of the disturbance in TRP- and TYR-associated NT metabolism caused by METH abuse and highlights NT metabolism as a promising therapeutic target for METH-induced neural and psychiatric disorders.

## 1. Introduction

Methamphetamine (METH), a representative of novel synthetic drugs, has emerged as one of the most prevalent, abused, and detrimental substances worldwide in recent years [1]. METH abuse induces extensive organ damage, with its prominent feature being high neurotoxicity that leads to profound impairments in learning, memory, and cognitive function, as well as the characterized psychiatric disorders including aggression, depression, self-harm, and even suicide [2,3,4,5,6]. Neurotransmitters (NTs), NT precursors, and metabolites are widely distributed throughout the central and peripheral nervous systems and play pivotal roles in regulating emotions and cognitive functions and behaviors [7,8]. The addictive nature of METH is closely associated with its significant impact on NTs, particularly dopamine (DA), and norepinephrine (NE) modulation, which shapes neural circuits involved in reward processing and motivation [9]; meanwhile, serotonin (5-HT) insufficiency in the brain is vulnerable to depressive-like changes [10,11]. Therefore, investigating the overall pathway changes in NTs among METH abusers will provide more comprehensive knowledge of the NT metabolic process, which may offer novel therapeutic strategies and potential targets. 

As a precursor for NT synthesis, tryptophan (TRP) plays a crucial role in various biological processes, particularly in neuropsychiatric activities and emotional behaviors [12]. Our previous metabolomic study revealed a significant reduction in TRP levels in the serum of METH abusers [13,14], though the mechanisms underlying this change remain unclear. As an essential amino acid, TRP metabolism occurs through three key pathways: the serotonergic, kynurenic, and microbial pathways, all of which play significant roles in regulating mood and cognition. Decreased TRP levels in the brain have been strongly linked to depression [15,16] and TRP or 5-Hydroxytryptophan (5-HTP) supplementation has been shown to alleviate depressive symptoms in animal models [17,18]. In addition, the serotonergic pathway is pivotal in cognitive functions, and the aberrant 5-HT levels contribute to memory impairments and decision-making difficulties [19,20]. For the kynurenic pathway, it is the primary metabolic pathway of TRP and produces both neuroprotective and neurotoxic metabolites [21], which are implicated in neurodegenerative diseases. For instance, kynurenic acid (KA) levels are significantly reduced in the brains of transgenic mouse models of Alzheimer’s disease (AD) [22], which is consistent with the declined levels of KA in plasma and erythrocyte of AD patients [23]. Additionally, recent studies have emphasized the importance of the gut–brain axis, highlighting the role of microbial metabolism in TRP pathways and its potential link to neurological and mental disorders. To date, research on addictive substances such as METH, heroin, morphine, and alcohol has predominantly focused on the central nervous system, circulatory system, and respiratory system, with limited attention given to the digestive system, particularly gut microbiota. Notably, studies have indicated that METH exposure alters the α and β diversity of mouse intestinal microbiota, leading to gut microbiota dysbiosis [24]. An analysis of the microbial profile of the METH-induced Conditioned Place Preference (CPP) model in rats through 16S rRNA gene sequencing revealed a significant increase in microbial diversity in the CPP group [25]. Furthermore, alterations in the gut microbiota abundance in METH-exposed mice were significantly associated with depressive-like behavior and neurotoxicity [26]. The TRP metabolites of the gut microbiota are crucial signaling molecules in the interaction between microbial communities and host–microbe relationships. Of note, the indole derivatives are the main metabolites of the TRP microbial metabolic pathway. The supplementation of indole and its derivatives effectively improves synaptic plasticity and ameliorates neuroinflammation in the AD model mice [27]. In clinical studies, specific levels of indole and its derivatives are closely associated with the severity of depressive and anxiety symptoms. Interestingly, the supplementation of indole-generative probiotics in elderly individuals leads to an increased serum indole-3-propionic acid (IPA), positively correlated with brain-derived neurotrophic factor levels [28]. However, the mechanisms underlying TRP metabolic pathway disturbances caused by METH remain unclear, and a full knowledge of the overall changes in TRP’s metabolic pathways is imperative. 

Therefore, in the current study, a UHPLC-MS/MS assay was employed to compare the NTs alterations in the serum of the METH abusers and the healthy controls specifically in the serotonergic, kynurenic, and microbial metabolic pathways, aiming to identify potential biomarkers or potential therapeutic targets for METH-associated neurological and psychiatric disorders.

## 2. Materials and Methods

### 2.1. Patient Population and Serum Sample Collection

This study employed a rigorous cross-sectional design, recruiting a total of 78 METH abusers (59 males and 19 females) and 79 carefully matched healthy controls (61 males and 18 females). The METH addicts were selected based on their well-documented and unequivocal history of METH abuse, ensuring the validity and reliability of the sample population. Stringent exclusion criteria were implemented to enhance the internal validity of the study, including the exclusion of individuals with acute psychosis, a history of detoxification treatment, severe organic diseases, and infectious diseases. Through implementing these stringent criteria, this study aimed to ensure that the observed effects were specifically attributable to METH abuse rather than confounding factors. The control group consisted of individuals without a history of drug abuse or significant organic disorders. They met stringent criteria, including no prior use of medications, psychotherapy, or other treatments that could influence the neurochemical profiles under investigation. Controls were also screened to ensure no recent exposure to drugs, alcohol, or drug-associated cues within the week before the study, minimizing confounding effects. 

Every participant duly signed the informed consent forms for the sample study. This study was approved by the Medical Ethics Management Committee of Changshu Hospital, affiliated with Suzhou University (Changshu First People’s Hospital, Ethical Application and Approval 2016-01). 

### 2.2. Neurotransmitter Extraction

Neurotransmitters were extracted from human serum, as described in previous studies, with some optimization [29,30]. Briefly, serum samples were thawed at room temperature, and 20 μL EDTA solution and 10 μL methanol mixture containing 200 ng/mL internal standard solution was added to 80 μL of human serum in 1.5 mL Eppendorf tubes. The internal standard solution consisted of TRP-D5 and IAA-D5, purchased from Toronto Research Chemicals (Toronto, ON, Canada). The samples were vigorously vortexed for 60 s and then precipitated on ice at 4 °C for 20 min. Subsequently, the samples were transferred to Nanosep 0.2 mM centrifugal devices and centrifuged at 8000× *g* for 15 min at 4 °C to remove insoluble matter, such as proteins. The resulting supernatant was carefully collected and injected into the UHPLC-MS/MS system for further analysis.

This standardized extraction procedure ensures the efficient isolation of neurotransmitters from human serum while minimizing interference from proteinaceous components and other insoluble matter. The use of an internal standard solution aids in the accurate quantification of neurotransmitter levels. The extracted supernatant is subsequently subjected to UHPLC-MS/MS analysis, enabling precise and sensitive measurements of the targeted neurotransmitters.

### 2.3. UHPLC-MS/MS Analysis

UHPLC-MS/MS analysis of human serum samples was conducted as previously described [31]. The samples were measured using the ExionLC™ AC HPLC system coupled with the SCIEX Triple Quad 5500 Plus mass spectrometer (AB SCIEX, Boston, MA, USA). The chromatographic separation of neurotransmitter extraction was achieved using the Acquity UPLC HSS T3 column (100 × 2.1 mm, 1.8 μm, Waters, Milford, MA, USA) at a flow rate of 0.4 mL/min, with an injection volume of 10 μL. The column was thermostated at 40 °C. The mobile phase consisted of solvent A (0.1% formic acid in water) and solvent B (0.1% formic acid in ACN). The gradient started and was maintained at 1% B for 0.5 min, then increased to 70% B within 1.5 min, further increased to 95% B within 2 min, and held for 1 min. The total runtime was 5 min, with a flow rate of 0.4 mL/min. A 2-min equilibration period was implemented before each subsequent injection.

The mass spectrometer was operated in multiple-reaction monitoring (MRM) mode with both positive and negative electrospray ionization. Key operating parameters included a source temperature of 150 °C, desolvation temperature of 450 °C, nitrogen gas flow rate of 900 L/h, cone voltage of 20 V, and capillary voltage of 2.50 kV. MS/MS conditions, such as precursor and product ion pairs, collision energy (CE), and declustering potential (DP), were optimized using 41 authentic standards. These standards were employed to identify the optimal CE and DP values for each neurotransmitter and metabolite, ensuring maximum sensitivity and specificity. To account for potential matrix effects and improve quantification accuracy, internal standards (Trp-D5 and IAA-D5) were added to each sample. These internal standards allowed the normalization of variations caused by sample matrices during analysis. Quantification was achieved using calibration curves constructed with authentic standards for each analyte, ensuring precise and reliable measurements.

### 2.4. Neurotransmitter Analysis

Peak extraction and correction were performed using the MultiQuant Version 3.0 (AB SCIEX, Framingham, MA, USA). The peak data were used to obtain endogenous metabolic material data. Before statistical analysis, the data were normalized through logarithmic transformation. MetaboAnalyst version 5.0 was utilized for data analysis. In cases where any sample had missing values, they were replaced with the limit of detection (LoDs), which were set at 1/5 of the minimum positive value for each variable.

### 2.5. Multivariate Analysis

Unpaired *t*-tests were conducted to compare the differences in demographic information between the groups. PCA, PLS-DA, and Orthogonal Partial Least Squares Discriminant Analysis (OPLS-DA) were performed using the MetaboAnalyst platform (https://www.metaboanalyst.ca/ accessed on 23 April 2023). To ensure the predictive ability of the OPLS-DA model and prevent overfitting, a permutation test with a permutation number of *n* = 100 was conducted. Neurotransmitters with a variable importance of projection (VIP) score > 0.8, fold change (FC) > 1.5 or < 0.667, and *p* value < 0.05 were considered differentially expressed in the serum between METH abusers and controls. Volcano plots were constructed using the Log2 (FC) and *p* value of the neurotransmitters from the Painuosen Genescloud platform (https://www.genescloud.cn accessed on 11 May 2023.) to visualize all detected neurotransmitters in human serum. The absolute concentrations of serum neurotransmitters and gender-specific heatmaps were analyzed using the Painuosen Genescloud platform. All heatmaps were generated using normalized auto-scaled data clustered by Euclidean distance and Ward’s linkage. R and SPSS were used for statistical analysis. The differences in serum neurotransmitters between METH abusers and controls were assessed using unpaired *t*-tests, and *p* values < 0.05 were considered statistically significant.

## 3. Results 

### 3.1. Analysis of Blood Biochemical Indexes and Multivariate Statistical Analysis of NTs and Metabolites in Serum

Blood and serum samples were collected from 78 METH abusers and 79 healthy controls. As shown in Table 1, METH abusers exhibited a significant increase in white blood cells (WBCs), platelets (PLTs), neutrophil count (NEUT), and fasting blood glucose (FBS) compared to the control group. Conversely, hemoglobin (Hb), lymphocyte (LYMPH), alanine aminotransferase (ALT), aspartate aminotransferase (AST), total bilirubin (Tbil), creatinine (Cr), blood urine nitrogen (BUN), retinol-binding protein (RBP), and total cholesterol (CHOL) were significantly decreased (*p* < 0.005) in METH abusers. In parallel, red blood cells (RBCs), eosinophil (EO), basophils (BA), direct bilirubin (Dbil), and triglyceride (TG) exhibited no significant changes between these two groups (*p* > 0.05). 

**Figure 1:**
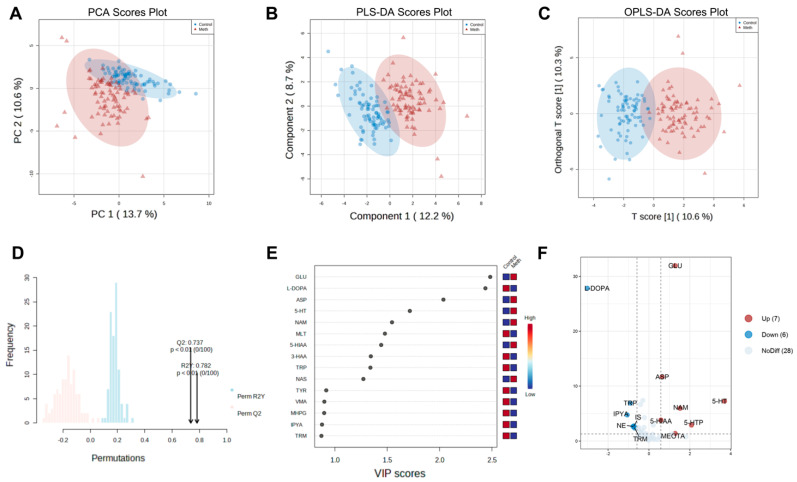
PCA of the NTs and their metabolites in the serum of METH abusers and healthy controls. (**A**) Nonsupervised PCA score plot. (**B**) PLS-DA score plot. (**C**) OPLS-DA score plot. Red triangles represent the METH abusers’ serum metabolomes; blue dots represent the healthy controls’ serum metabolomes. (**D**) Permutation test. It verifies the prediction ability of the OPLS-DA model. *p* values of <0.05 were considered statistically significant. The permutation number was set to 100. (**E**) VIP plot of the serum metabolomes. VIP > 0.8. (**F**) Volcano plot. Red dots: upregulated metabolites; blue dots: downregulated metabolites; gray dots: no obviously affected metabolites.

Given the close linkage between inflammation and the disturbed NTs, a total of 41 NTs (including TRP and tyrosine (TYR)-derived metabolic NTs) were detected in the serum by the UHPLC-MS/MS assay. PCA and PLS-DA demonstrated a distinct separation between serum samples of METH abusers and controls, highlighting significant alterations in NT and metabolite profiles associated with METH exposure (Figure 1A, B). The OPLS-DA model, a supervised analysis technique with strong discriminatory power, successfully segregated serum samples from different groups (R2Y = 0.782 and Q2 = 0.737). To avoid overfitting, 100 permutation tests were conducted (Figure 1C,D). Inconsistencies were observed in NTs between METH abusers and the control samples. Significant altered NTs and metabolites, with variable VIP scores > 0.8 and FC > 1.5 or < 0.667, meeting the *t*-test at the 95% confidence level, are listed (Figure 1E,F).

### 3.2. Analysis of Differential NTs and Neuroactive Metabolites in the TRP Pathway

**Figure 2:**
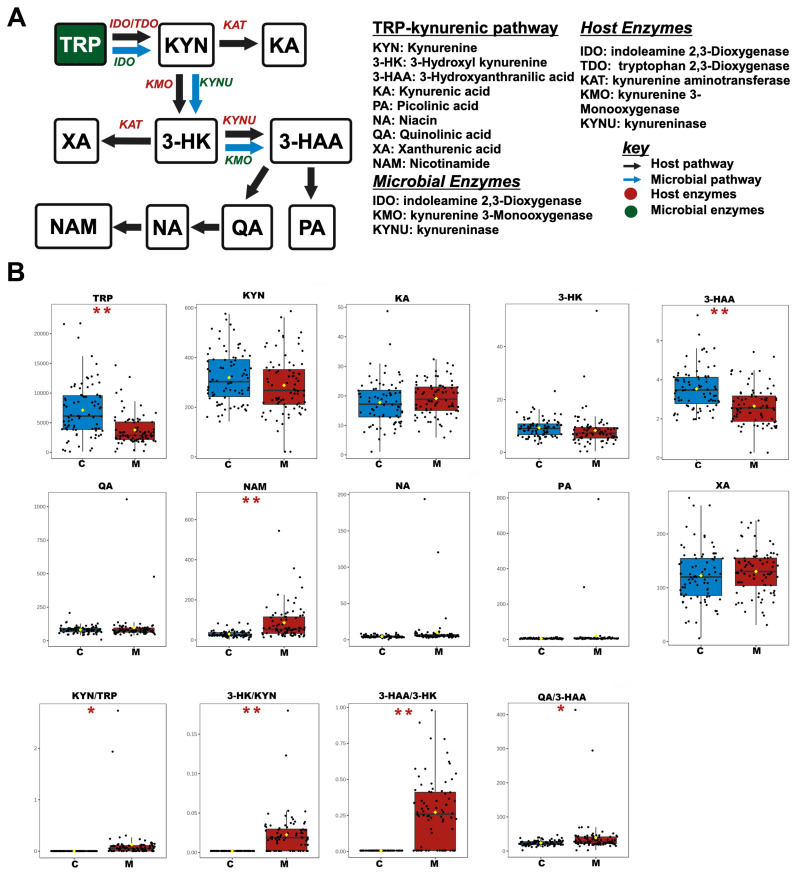
The changed serum NTs and the dynamic metabolism of the TRP–kynurenic pathway in METH abusers. (**A**) Pathway schematic of TRP–kynurenic metabolism. Black arrows show the host pathway; blue arrows show the microbial pathway. Host enzymes with genomic evidence are marked in red; microbial enzymes with genomic evidence are marked in green. (**B**) Differences in absolute concentrations (ng/mL) of TRP–kynurenic metabolites and the statistically significant ratios in the TRP–kynurenic pathway. C, healthy controls. M, METH abusers. * *p* < 0.05; ** *p* < 0.01.

**Figure 3:**
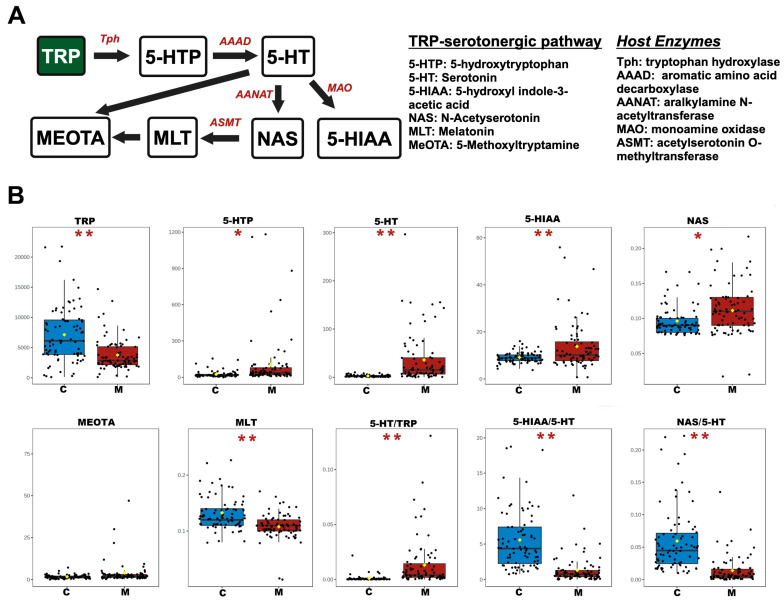
The changed serum NTs and the dynamic metabolism of the TRP–serotonergic pathway in METH abusers. (**A**) Pathway schematic of TRP–serotonergic metabolism. Black arrows indicate the host degradation pathway; host enzymes with genomic evidence are marked in red. (**B**) Differences in absolute concentrations (ng/mL) of TRP–serotonergic metabolites and the statistically significant ratios in the TRP–serotonergic pathway. C, healthy controls. M, METH abusers. * *p* < 0.05; ** *p* < 0.01.

**Figure 4:**
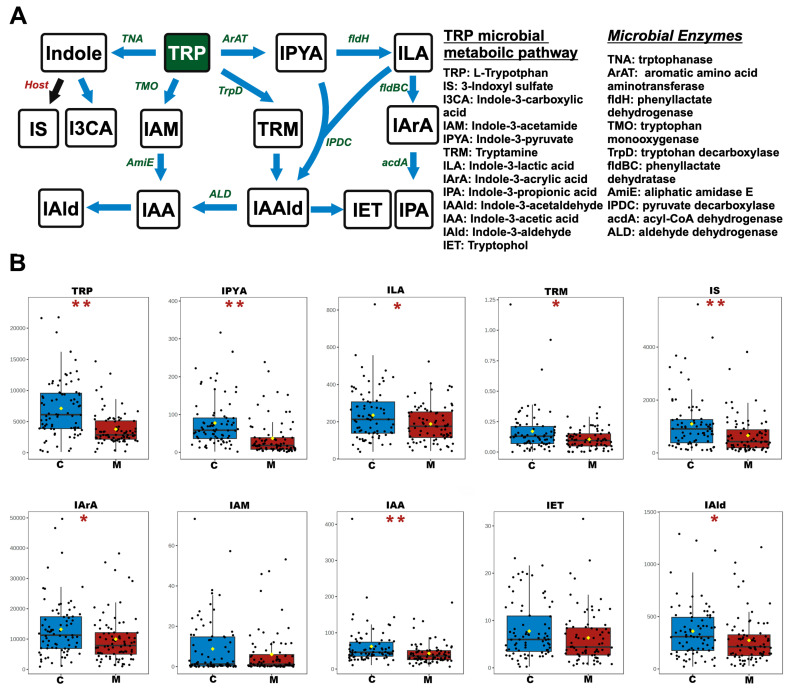
The changed serum NTs and the dynamic metabolism of the TRP–microbial metabolic pathway in METH abusers. (**A**) Pathway schematic of TRP–microbial metabolism. Black arrows show the host pathway; blue arrows show the microbial pathway. Host enzymes with genomic evidence are marked in red; microbial enzymes with genomic evidence are marked in green. (**B**) Differences in absolute concentrations (ng/mL) of TRP–microbial metabolism and the statistically significant ratios in the TRP–microbial metabolic pathway. C, healthy controls. M, METH abusers. * *p* < 0.05; ** *p* < 0.01.

Our previous results showed a marked reduction in TRP levels in the serum of METH abusers [13,14], so we sought to examine how METH abuse affects TRP metabolism. Therefore, the metabolic pathways of the TRP, including the kynurenic pathway, serotonergic pathway, and microbial metabolic pathway, were investigated. For the kynurenic pathway, discernible alterations were observed in METH abusers compared to the control group (Figure 2). A notable reduction in 3-hydroxyanthranilic acid (3-HAA) levels, along with a significant increase in nicotinamide (NAM) concentrations, was observed in the serum of METH abusers. Additionally, key ratios including kynurenine (KYN)/TRP, 3-hydroxykynurenine (3-HK)/KYN, 3-HAA/3-HK, and quinolinic acid (QA)/3-HAA exhibited a substantial increase, indicating the conversion of the kynurenic pathway to a toxic metabolic pathway. For the serotonergic pathway, the levels of 5-HTP, 5-HT, 5-hydroxyl indole-3-acetic acid (5-HIAA), and n-acetyserotonin (NAS) in the serum of METH abusers were significantly increased (Figure 3), while melatonin (MLT) concentrations were significantly decreased. Concurrently, the key ratios of 5-HT/TRP were significantly increased, while the 5-HIAA/5-HT and NAS/5-HT ratios were decreased, emphasizing the amelioration of the protective effects in this process. For the microbial metabolic pathway, a remarkable reduction in indole-3-pyruvate (IPYA), indole-3-lactic acid (ILA), tryptamine (TRM), 3-indoxyl sulfate (IS), indole-3-acrylic acid (IArA), indole-3-acetic acid (IAA), and indole-3-aldehyde (IAld) in the serum of METH abusers was observed when compared to the controls (Figure 4). These results, taken together, indicate the deteriorated effects of METH abuse in the TRP metabolic pathway. 

### 3.3. Analysis of Differential NTs and Neuroactive Metabolites in the TYR–Dopamine Pathway and Other Amino Acid Neurotransmitters

**Figure 5:**
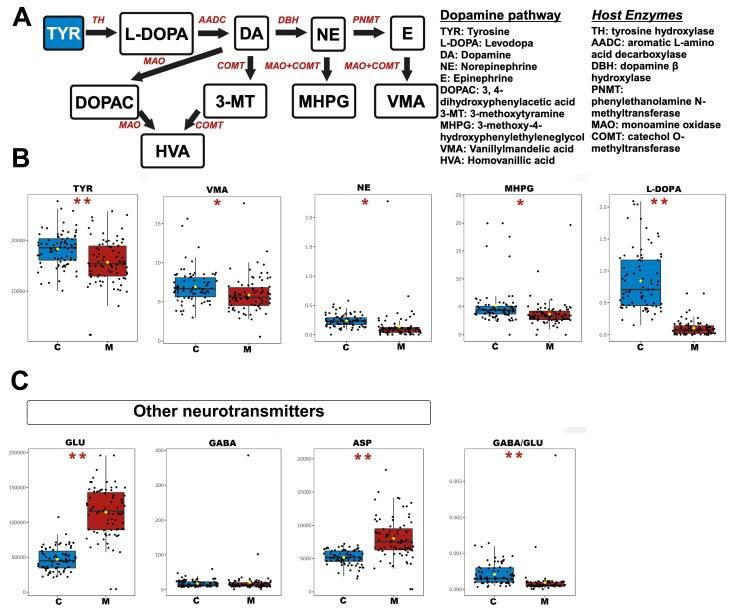
The changed serum NTs and the dynamic metabolism of the TYR–dopamine pathway in METH abusers. (**A**) Pathway schematic of TYR–dopamine metabolism. Black arrows indicate the host degradation pathway; host enzymes with genomic evidence are marked in red. (**B,C**) Differences in absolute concentrations (ng/mL) of TYR–dopamine metabolism, other amino acid NTs, and the statistically significant ratios. C, healthy controls. M, METH abusers. * *p* < 0.05; ** *p* < 0.01.

Due to the critical roles of dopamine in mental modulation, the TYR–dopamine metabolic pathway was dissected. As depicted in Figure 5, the metabolites in the serum of METH abusers exhibited distinct alterations compared to the control group. In particular, the concentrations of TYR, vanillylmandelic acid (VMA), NE, 3-methoxy-4-hydroxyphenylethyleneglycol (MHPG), and levodopa (L-DOPA) were decreased in METH abusers. For other amino acid neurotransmitters, the levels of glutamate (GLU) and aspartic acid (ASP) in the serum of METH abusers increased concomitantly with a decreased γ-Aminobutyric acid (GABA)/GLU ratio, while the levels of GABA and glycine (GLY) displayed no obvious alterations. 

### 3.4. The Predictive Potential of the Neurotransmitters in Distinguishing METH Abusers

**Figure 6:**
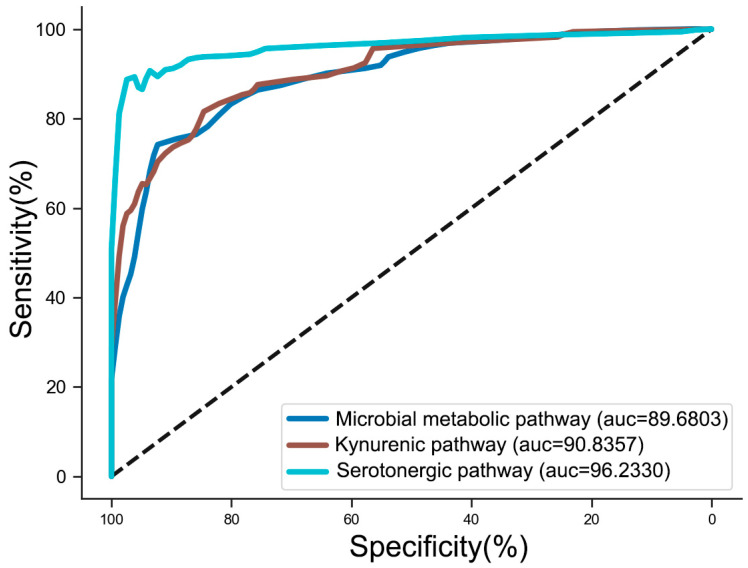
ROC curves distinguishing the controls and the METH abusers. Model 1 contained the TRP–microbial metabolic pathway. Model 2 contained the TRP–kynurenic pathway. Model 3 contained the TRP–serotonergic pathway.

To determine the predictive potential of these neurotransmitters in differentiating groups, cross-validated models were built. The receiver operating characteristic (ROC) curves of selected metabolites of the three TRP pathways are presented in Figure 6. Model 1, which contained metabolites from the microbial metabolic pathway (IAA, TRM, Tryptophol (IET), IPA, IAld, Indole-3-acetamide (IAM), IArA, Indole-3-lactic acid (ILA), IPYA, IS), achieved an area under the ROC curve (AUC) of 0.8968. Model 2, which included neurotransmitters from the kynurenic pathway (KYN, NAM, KA, Picolinic acid (PA), Niacin (NA), Xanthurenic acid (XA), QA, 3-HAA, 3-HK), achieved an AUC of 0.9084. Model 3, which included neurotransmitters from the serotonergic pathway (Methoxytryptamine (MEOTA), 5-HTP, 5-HIAA, 5-HT, MLT, NAS), achieved an AUC of 0.9623.

### 3.5. Gender-Specific Changes in Serum NTs and Neuroactive Metabolites Between METH Abusers and Controls

Clinical studies have shown that men and women respond differently to psychostimulants, including METH [32,33]. Previous research has also alluded to gender-specific variations in the metabolites induced by METH in both genders [13]. However, a gender-specific comparison of NT disparities from TRP metabolic pathway between METH abusers and healthy individuals is unknown.

**Figure 7:**
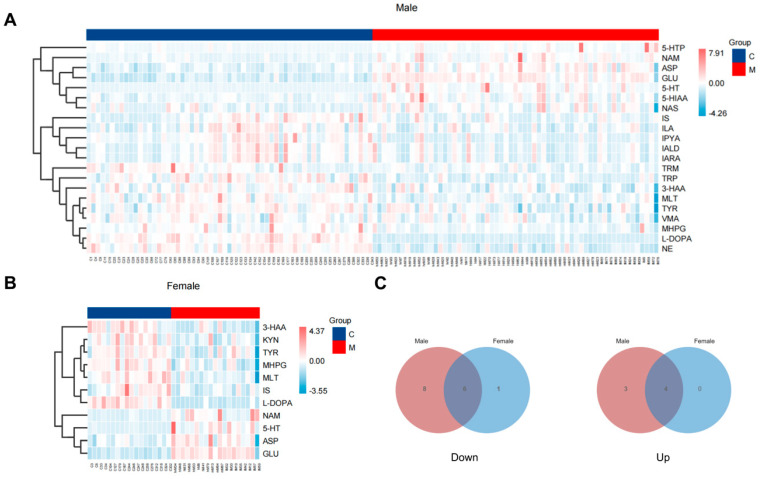
Alterations in NTs and neuroactive metabolites from the *t*-test in METH abusers of different genders. (**A,B**) Heatmaps of statistically significant metabolites from the *t*-test in male and female METH abusers. C, healthy controls. M, METH abusers. hrM, METH abusers of higher exposure. hM, METH abusers of high exposure. mM, METH abusers of medium exposure. lM, METH abusers of low exposure. (**C**) Venn plot that illustrates differences in serum metabolites between male and female METH abusers, with overlapping areas indicating shared changes caused by METH use in both genders.

Based on the findings presented in Figure 7 and Table 2, compared to the control group, 14 NTs and neuroactive metabolites decreased, while 7 NTs increased in male METH abusers; in females, 7 NTs and neuroactive metabolites declined, whereas 4 NTs increased. A significant decrease in IS in the serum of both males and females in the TRP–microbial metabolic pathway was observed. Notably, the levels of indole derivatives produced by the gut microbiota were almost reduced in male METH abusers. In the TRP–serotonergic pathway, a pronounced elevation of the metabolites across the entire pathway in males was observed, while in females, only the 5-HT significantly increased. For the TRP–kynurenic pathway, only the KYN levels in females exhibited a significant reduction, while for the TYR–dopamine pathway, the declined levels of NE and VMA were observed only in males. These results revealed the intricate differences in TRP-derived metabolites between males and females.

## 4. Discussion

Methamphetamine (METH) abuse triggers profound psychiatric and neurological disorders, including depression and anxiety, which are closely linked to disturbance in NT metabolism. Although the neurotoxicity of METH has been widely studied, its systemic effects on TRP-associated metabolic pathways remain poorly understood. Thus, the present study provides a comprehensive analysis of 41 NTs, precursors, and metabolites across the serotonergic, kynurenic, and microbial metabolic pathways in the serum of METH abusers, aiming to elucidate the underlying mechanisms of METH-induced psychiatric disorders and identify potential therapeutic targets.

Not limited to the decreased levels of TRP in our previous studies [13,14], the current work expanded the discovery of the alterations in TRP metabolism involving the kynurenic, serotonergic, and microbial pathways in METH abusers, which provided a more detailed analysis of how METH disturbs NT metabolism via TRP- and TYR-associated pathways, which may offer clues of METH-induced psychiatric and neurological disorders.

The kynurenic pathway is gaining more attention for its critical roles in psychiatric, hepatic, and immunological disorders [21]. Indoleamine 2,3-dioxygenase (IDO) and tryptophan 2,3-dioxygenase (TDO) are the two main rate-limiting enzymes responsible for the conversion of TRP into KYN, which then primarily metabolizes along two branches (Figure 2A), the neuroprotective metabolite KA, or the neurotoxic metabolites 3-HK and QA [34]. The dynamic conversion of TRP from 5-HT to KYN, as evidenced by an increased KYN/TRP ratio, is found in depression, which heavily depends on the activation of IDO or TDO enzymes. Intriguingly, the significant increase in the ratios of 3-HK/KYN and 3-HAA/3-HK in the current work implied the METH-driven toxic pathways, which are closely associated with kynureninase (KYNU) and kynurenine 3-monooxygenase (KMO) activation or upregulation, and these rate-limiting enzymes are required to validate in METH treatment mice in future work. Moreover, other key enzymes such as IDO, TDO, and KMO must also be detected to better understand their roles in the METH-induced disturbance of the kynurenic pathway. Interestingly, through gender-specific analysis, a decrease in KYN levels in female abusers was observed. This finding may suggest potential differences in downstream TRP metabolism between males and females. However, the specific roles of rate-limiting enzymes such as KMO and KYNU in this gender-specific difference remain unclear and require further investigation. These differences might partially account for the vulnerability of females to depression, and more studies are needed to confirm this association [35]. NAM is the end product of the KYN metabolism and a precursor for NAD^+^. NAD^+^ plays a crucial role in maintaining cellular redox balance. In the salvage synthesis pathway of NAD^+^, Nicotinamide phosphoribosyltransferase (NAMPT) acts as the rate-limiting enzyme [36,37]. In the current study, NAM was increased in the METH abusers, which may be ascribed to the aberrant expression of NAMPT since the administration of NAM to mice does not effectively increase NAD^+^ levels [38], posing a critical role of NAMPT in maintaining NAD^+^ homeostasis. On the contrary, high doses of NAM can lead to adverse effects such as insulin resistance, Parkinson’s disease, and hepatic toxicity [39,40,41]. The elevated levels of NAM in our study may suggest a potential link to METH’s impact on cellular energy metabolism since lacking NAD^+^ severely affects mitochondrial respiration and its ability to provide energy. Nonetheless, the precise effects and the underlying mechanisms involving METH-induced NAD^+^ metabolism are unknown, and further research should focus on this enzyme’s role in METH-induced NAD^+^ dysregulation. In addition, in the current work, the marked increased ratios of QA/3-HAA and 3-HAA/3-HK demonstrate the toxic conversion of KYN to 3-HK and QA rather than the neuroprotective substance KA.

Indole derivatives are the primary metabolic products in the TRP–microbial metabolic pathway, and the gut microbiota can directly metabolize TRP into several indole derivatives such as IAld, IAA, IPA, IPYA, etc. Intriguingly, a striking reduction in serum IPYA, ILA, TRM, IS, IArA, IAA, and IAld in METH abusers was observed compared to the control group, suggesting the disturbance of the TRP–microbial metabolic pathway in the serum of METH abusers. Recent studies have demonstrated that METH-treated mice exhibit significantly reduced beneficial genera *Akkermansia* [42], which is closely associated with indoles and TRP derivative production [43]. These results supported the potential roles of METH in intestinal microbiota dysbiosis. However, no data are available on the issue of intestinal microbiota between male and female METH abusers. Of note, the levels of indole derivatives produced by microbiota in males were almost uniformly decreased in the current work, suggesting that some of the specific microbiota responsible for TRP metabolism were more prone to METH challenge in males. However, the lack of intestinal microbiome analysis in this study limits our understanding of the specific microbial species involved in these alterations, and the microbial sequencing results could complement future work. 

The serotonergic pathway is another branch of the TRP metabolic pathway and plays a key role in emotional and mental behaviors [44,45]. It is well known that amphetamine-like drugs cause a substantial release of 5-HT, resulting in a brief euphoric sensation. Consistent with this phenomenon, our study revealed a significant elevation of 5-HTP, 5-HT, NAS, and 5-HIAA in the serum of METH abusers, concomitant with the increased 5-HT/TRP ratio. Reciprocally, a decreased level of MLT, an NT potentially associated with circadian rhythms and sleep regulation, was found; this phenomenon may be ascribed to the decreased ratio of NAS/5-HT, since the NAS serves as a precursor of melatonin. Interestingly, in a gender-specific analysis, an overall increase in the metabolites of the serotonergic pathway in male METH abusers was observed. In contrast, the females showed elevated levels of 5-HT only, which was in line with the gender-specific metabolism of METH abuse in our previous study [13]. Direct conversion from 5-HT to downstream decreased since the level of MLT decreased. Despite these findings, it remains unclear whether METH directly influences enzymes regulating serotonin metabolism, such as tryptophan hydroxylase (TPH) or serotonin N-acetyltransferase. Future studies should include enzyme-specific analyses to clarify the mechanisms underlying these metabolic shifts.

The TYR pathway is crucial in NT metabolism, involved in synthesizing monoamine NTs (DA, NE, and E), which play key roles in maintaining physiological activities such as emotions and behaviors [46]. In the present study, TYR was decreased in METH abusers, accompanied by reductions in their metabolites, including L-DOPA, NE, MHPG, and VMA. In the gender-specific analysis, a significant decrease in NE and VMA levels was observed only in male abusers. This finding suggests potential sex-specific differences in TYR metabolism. However, the roles of enzymes such as monoamine oxidase (MAO), catechol-O-methyltransferase (COMT), and dopamine β-hydroxylase (DBH) in this process remain unclear and require further investigation. The smaller number of female samples in this study limits the statistical power of these observations, and increasing the number of female participants in future studies would improve the reliability of gender-specific analysis. For other amino acid metabolism, excitatory (GLU and ASP) and inhibitory NTs (GLY and GABA) were examined. GABA is generated from GLU catalyzed by glutamate decarboxylase (GAD) [47]. Numerous studies have indicated a significant increase in GLU and ASP in the brains of animals administered METH or in the serum of METH abusers [13,14,48], which may partially delineate METH-induced neural damage. Additionally, the cross-validated models using all substances from the three metabolic pathways of TRP were performed. The AUC of the serotonergic pathway model reached 0.9623, which may serve as a potential indicator for METH abuse. However, these findings require validation in larger and more diverse populations, as well as in animal models of METH abuse. Such models would allow for the controlled exploration of rate-limiting enzymes and behavioral correlations, further elucidating the role of TRP metabolism in METH-induced disorders.

## 5. Conclusions

This study demonstrates that METH abuse induces significant disruptions in TRP-associated neurotransmitter metabolism across the kynurenic, serotonergic, and microbial pathways. Key findings include a toxic shift in the kynurenic pathway, reductions in microbial metabolism-related indole derivatives, and altered serotonin metabolism characterized by elevated 5-HT and reduced MLT. These results highlight METH’s systemic effects on TRP metabolism, which may contribute to psychiatric symptoms and neurological damage. Moreover, the gender-specific differences in TRP metabolism are another discovery in the present work, further underscoring the complexity of METH-induced metabolic disturbance. While the reduction in microbial metabolism-related indole derivatives suggests potential gut microbiota dysbiosis, the lack of direct microbiota data limits definitive conclusions. Future research should focus on integrating gut microbiota analysis with enzymatic and metabolomic profiling to better understand the role of TRP metabolism in METH abuse. 

## Figures and Tables

**Table 1:** Analysis of blood biochemical indexes between METH abusers and controls *.

	Controls (*n* = 79)	METH Abusers (*n* = 78)	*p* Value
Age (years)	38.10 ± 8.68	38.09 ± 8.79	0.9632
Gender (*n*)			
Male	61	59	—
Female	18	19	—
Biochemical indexes			
RBC (×10^12^/L)	4.95 ± 0.39	4.81 ± 0.48	0.0697
Hb (g/L)	148.9 ± 13.87	140.4 ± 19.48	0.0032
WBC (×10^12^/L)	6.86 ± 1.51	8.51 ± 2.31	<0.0001
PLT (×10^9^/L)	209.8 ± 64.33	260.87 ± 66.88	<0.0001
NEUT (%)	55.9 ± 6.34	68.39 ± 8.41	<0.0001
LYMPH (%)	36.01 ± 6.1	24.96 ± 7.25	<0.0001
EO (%)	2.31 ± 1.85	2.02 ± 1.26	0.2922
BA (%)	0.38 ± 0.24	0.33 ± 0.14	0.1827
ALT (U/L)	29.94 ± 23.25	19.81 ± 14.03	0.0014
AST (U/L)	22.38 ± 7.610	18.16 ± 8.91	0.0018
BUN (mmol/L)	5.4 ± 1.08	4.24 ± 1.52	<0.0001
FBS (mmol/L)	5.27 ± 0.75	6.51 ± 2.30	<0.0001
Tbil (μmmol/L)	15.63 ± 5.65	11.66 ± 4.77	<0.0001
Dbi (μmmol/L)	4.92 ± 1.91	4.62 ± 1.99	0.3412
Cr (μmmol/L)	73.05 ± 15.72	59.91 ± 13.71	<0.0001
RBP (μg/ml)	52.15 ± 10.25	34.40 ± 10.83	<0.0001
TG (mmol/L)	1.66 ± 0.83	1.48 ± 0.80	0.1957
CHOL (mmol/L)	4.98 ± 0.77	4.17 ± 0.89	<0.0001

* Data are mean ± SD. *p* values for comparison between measurements of controls and METH abusers. RBC, red blood cell; Hb, hemoglobin; WBC, white blood cell; PLT, platelet; NEUT, neutrophil; LYMPH, lymphocyte; EO, eosinophil; BA, basophils; ALT, alanine aminotransferase; AST, aspartate aminotransferase; BUN, blood urine nitrogen; FBS, fasting blood glucose; Tbil, total bilirubin; Dbil, direct bilirubin; Cr, creatinine; RBP, retinol-binding protein; TG, triglyceride; CHOL, total cholesterol.

**Table 2:** Gender-specific changes in serum NTs and neuroactive metabolites between METH abusers and controls.

Gender	Metabolites	FC	VIP	*p* Value	Changes ^a^
Male	5-HT	14.019	1.555	<0.001	up
	GLU	2.345	2.440	<0.001	up
	NAM	2.891	1.434	<0.001	up
	ASP	1.622	2.036	<0.001	up
	5-HIAA	1.502	1.517	<0.001	up
	5-HTP	3.800	<0.8	<0.001	up
	NAS	0.667 < FC < 1.5	1.199	<0.001	up
	TRP	0.474	1.456	<0.001	down
	TRM	0.553	1.003	<0.01	down
	L-DOPA	0.123	2.383	<0.001	down
	IPYA	0.414	0.995	<0.001	down
	NE	0.426	1.819	<0.001	down
	MLT	0.667 < FC < 1.5	1.281	<0.001	down
	3-HAA	0.667 < FC < 1.5	1.075	<0.001	down
	TYR	0.667 < FC < 1.5	<0.8	<0.01	down
	ILA	0.667 < FC < 1.5	<0.8	<0.05	down
	IArA	0.667 < FC < 1.5	<0.8	<0.05	down
	MHPG	0.667 < FC < 1.5	0.818	<0.05	down
	IAld	0.667 < FC < 1.5	<0.8	<0.05	down
	IS	0.667 < FC < 1.5	<0.8	<0.05	down
	VMA	0.667 < FC < 1.5	<0.8	<0.05	down
Female	GLU	2.884	2.273	<0.001	up
	5-HT	10.101	1.675	<0.01	up
	NAM	2.835	1.416	<0.01	up
	ASP	0.667 < FC < 1.5	1.514	<0.01	up
	L-DOPA	0.123	2.233	<0.001	down
	3-HAA	0.581	1.528	<0.001	down
	IS	0.438	1.447	<0.01	down
	MLT	0.667 < FC < 1.5	1.606	<0.001	down
	MHPG	0.667 < FC < 1.5	1.663	<0.001	down
	KYN	0.667 < FC < 1.5	1.249	<0.01	down
	TYR	0.667 < FC < 1.5	0.965	<0.05	down

Abbreviations: FC, fold change; VIP, variable importance of projection; ^a^ Up, upregulated metabolites in METH abusers versus controls in unpaired *t*-test. Down, downregulated metabolites in METH abusers versus controls in unpaired *t*-test.

## Data Availability

Data are available on request to the authors.
